# Schistosomiasis, hepatitis B and hepatitis C co-infection

**DOI:** 10.1186/s12985-015-0251-2

**Published:** 2015-02-08

**Authors:** Gasim I Gasim, Abdelhaleem Bella, Ishag Adam

**Affiliations:** Qassim College of Medicine, Qassim University, Buraydah, Saudi Arabia; King Fahad Hospital of the University, University of Dammam, Dammam, Saudi Arabia; Faculty of Medicine, University of Khartoum, P.O. Box 102, Khartoum, Sudan

**Keywords:** Schistosomiasis, HBV, HCV, Hepatotropic, Vaccine, Adjuvant

## Abstract

**Background:**

Schistosomiasis is a significant health problem in more than 70 countries distributed between Africa, Asia and South America, with an infection rate of one in 30 individuals. Data on Schistosomiasis, Hepatitis B virus (HBV) and Hepatitis C virus (HCV) co-infection are scarce; however, there is a high prevalence in countries where schistosomiasis is endemic.

**Methods:**

A systematic search was performed on published data from 1980–2014. Published papers in the databases Google, Medline, PubMed, and MiPc library were searched using the keywords epidemiology, pathogenesis and outcomes of HBV, HCV and schistosomiasis and data were extracted from the relevant studies.

**Results:**

The prevalence of HBV/schistosomiasis co-infection in countries where schistosomiasis is endemic was high, ranging between 9.6 to approximately 64% in Egypt, and a maximum of 15.8% among hospitalized patients in Brazil. Concurrent infection between HBV and schistosomiasis is often associated with countries where schistosomiasis is endemic and may lead to chronic liver inflammation. Similarly, HCV infection rates in schistosomiasis populations range from 1% in Ethiopia reaching up to 50% in Egypt.

**Conclusion:**

There is controversy regarding the effects of HBV and HCV on schistosomiasis and vice versa. Vaccination might be a solution to the era of schistosomiasis and co-infection with HBV and HCV.

## Introduction

Schistosomiasis is a parasitic infection that is second to malaria in prevalence and affects about 200 million people in over 70 countries with an infection rate of one in 30 individuals [1,2]. It is of particular importance in Africa and South America owing to its endemicity, high prevalence and morbidity rates in countries such as Nigeria, Tanzania, Democratic Republic of Congo, Ghana, and Brazil [1,3]. The prevalence of schistosomiasis was estimated to range between 3 to 10% in Egypt while in Tanzania it ranges from 2.7% to 35.6% [4,5]. Schistosomiasis is a complex tropical disease caused by Schistosoma species, of which *Schistosoma haematobium, S. mansoni, and S. japonicum* are the most common [6]. The diverse clinical patterns of this infection depend on the interplay of numerous factors, such as parasite strain, host genetic background, host immunity and nutritional status, and co-infections [7-11]. Often, disease progresses to an advanced stage, called hepatosplenic schistosomiasis (HSS), which is frequently seen in endemic areas and is characterized by portal hypertension that may lead to gastrointestinal bleeding [12,13].

Concurrent infection between hepatitis B virus (HBV) and schistosomiasis is often observed in countries where schistosomiasis is endemic and might cause chronic liver inflammation [14]. *S. mansoni* and HBV co-infection pathogenesis remains controversial; however, the harmful effects of *S. mansoni* and hepatitis C virus (HCV) co-infection on liver fibrosis have demonstrated in endemic countries [15-17]. Chronic HCV infection is a prevalent chronic liver disease globally, with a burden approaching of 185 million people testing positive for HCV antibodies (2.8% prevalence) with the highest prevalence in North and East Asia, the Middle East and North Africa region (>3.5% prevalence) [18]. The three infections (schistosomiasis, HBV, HCV) induce acute and chronic manifestations that mainly affect the liver with variable degrees and patterns of liver dysfunction. Therefore, co-infection may have an impact on disease pathophysiology which is assessed in this article. Treatment of any of the three conditions might impact on the other infection. For example, in Egypt, the use of unsterile syringes during mass campaigns of intravenous anti-schistosomiasis treatment was suggested to be the main reason for the spread of HCV [19]. This review was conducted to address the gap of knowledge regarding the above mentioned co-infections.

### Review

A systematic search of published data during the period 1980–2014was performed. Published papers on epidemiology pathogenesis and outcomes of HBV and schistosomiasis were extracted from relevant studies provided they are written in English or at least their abstracts are in English. Databases were searched using different search engines including Medline, PubMed, MiPc library and Google with the keywords “Hepatitis B virus”, “schistosomiasis”, “epidemiology”, “Africa”’ and “South America” and names of particular countries where they were entered interchangeably. We excluded articles using serological methods to diagnose schistosomiasis.

### Epidemiology of co-infection with schistosomiasis, HBV and HCV

Published data on HBV and schistosomiasis co-infection is scarce where we found about 15 articles fulfilling our criteria; however, in countries where schistosomiasis is endemic such as Egypt, a high prevalence of HBV and *S. mansoni* co-infection has been described ranging from 19.6 to 33.0% [20,21]. The prevalence of co-infection among the general population in Brazil is 15.8% [22]. A high (58.4%) prevalence of HBV was reported among Chinese patients with chronic schistosomiasis [23].

Similarly, published data about HCV and schistosomiasis co infection is scanty where only 16 articles could fulfill our criteria. Prevalence rates of HCV infection with wide variations as low as 1% and as high as 50% among patients with schistosomiasis, were reported in different countries [24-26]. Likewise high (40.2%) prevalence of HCV and *S. mansoni* antibodies were detected among 233 wastewater treatment plants workers [27]. Interestingly, a more recent study among 3,596 Egyptian patients found that 27.3% had both HCV-RNA and schistosomiasis, though a weak point is their reliance on serology to diagnose schistosomiasis, a thing that questions the association between HCV and schsitosomiasis [28]. Nonetheless, in Yemen a group of researchers reported a relationship between *S. haematobium* and hepatitis B, but no correlation between *S. mansoni* infection and HBV or HCV [29]. The prevalence of *S. mansoni* infection was 65.9% in a community-based study in Ethiopia including 2,451 subjects. Moreover, the prevalence of schistosomal periportal thickening/fibrosis (PPT/F) was 4.6%. At least one marker of either HBV or HCV was detected in 43.2% of the subjects, of which 5.3% were HBsAg positive, and 1.3% were anti-HCV [30] positive see Table 1. PPT/F increased significantly and proportionally in terms of prevalence with community prevalence and intensity of *S. mansoni* infection [30]. It is understandable that HSS renders patients at risk of getting HCV owing to the involvement with the risk factors for both HBV and HCV such as history of treatment with parenteral treatment for schistosomiasis unsterile syringes during mass campaigns, blood transfusion, surgical and endoscopic interventions [21,31-35].

**Table 1:** **Showing the prevalence of co-infection HCV/ HBV and hepatosplenic schistosomiasis (HSS)**

**Country**	**Prevalence of HCV/HSS**	**Remarks about the study**	**Prevalence of HBV/HSS**	**Remarks about the study**
Egypt	33% [21]	1-Angelico et al [21] a small scale study carried among 135 liver disease patients	19.6% [20]-33% [21]	1-El-Sayed et al [20]
				This is a community based study
				2-Angelico et al [21] a small scale study carried among 135 liver disease patients
		2- el-Esnawy [27] et al. carried a study among 233 waste water treatment workers		
	40.2% [27]			
Sudan	2.3%	Mudawi et al. [25] studied 176 schistosomiasis patients		
Ethiopia	4%	Berhe et al. [30] studied 199 schistosomiasis patients	16.1%	Berhe et al. [30] studied 199 schistosomiasis patients
Brazil	12.9%	Aquino et al [22] studied 101 patients with HSS	15.8%	Aquino et al [22] studied 101 patients with HSS
China	0.06%	Li et al. [26] studied 102 patients with schistosomiasis	58.4%	Du et al. [23] studied 250 schistosomiasis patients

### Immunology of schistosomiasis and hepatitis B or C co-infection

Wahib *et al.,* demonstrated the co-infection of HBV and/or HCV in 120 patients with hepatosplenic schistosomiasis was associated with a marked depression in cell mediated immune responses [36]. Furthermore, Edwards *et al.,* described the effects of hepatotropic virus co-infection during the Th2-dominated granulomatous phase of Schistosomal infection. They used lymphocytic choriomeningitis virus (LCMV) as a model for hepatotropic viruses, and demonstrated the induction of a strong LCMV-specific T cell response, with infiltration of large numbers of LCMV-specific interferon (IFN)-γ-producing CD8+ cells into the liver. This can lead to the down regulation of Th2 cytokine production that is dominant during *S. mansoni* infection and expeditious hepatotoxicity related morbidity. Moreover, livers of co-infected mice were highly susceptible to viral replication, which correlated with a reduction in intrahepatic type I IFN responses following virus infection [36-38].

HCV infection worsens the outcome of concomitant schistosomiasis and HCV infection, causing advanced liver disease, higher HCV RNA titers, an increase in histological activity index, increased incidence of liver cirrhosis and hepatocellular carcinoma, and increased mortality rate [17,39]. *S. mansoni* co-infection targets a specific subset of memory CD8+ T cells during HCV infection [40]. The immune response in *S. mansoni* patients with or without HCV infection was evaluated by assaying the serum levels of IFN-γ and IL-5 to estimate cell mediated immunity and IgE levels to estimate humoral immunity. A shift to T helper (Th)-0 and Th-2 immunity as well as an associated reduction of Th-1 responses( more or less a shift from cellular to humoral immunity) were observed, which might have a role in the persistence and severity of both diseases. Such immunity defects can lead to less HCV clearance [41,42]. *S. haematobium* soluble egg antigen (SEA) affects the intracellular HCV RNA burden in peripheral mononuclear cells (PBMC) as well as cell proliferation in patients with chronic HCV infection, which is linked, in part, to the direct triggering of viral replication by SEA [43]. Lymphotoxin (LT)-α contributes to inflammatory responses and single-nucleotide polymorphisms (SNP) in the human LT-α gene might have significant effects on individual susceptibility to disease. Individuals with HCV infection alone and those co-infected with schistosoma and HCV (but not those infected with Schistosoma alone) were significantly more likely to carry the LN-α gene mutation than the control subjects [44]. The common IL-10 (−1082, −819 and −592) genotypes/haplotypes were not associated with susceptibility to HCV infection either alone or during co-infection with *S. mansoni* [45].

### Impact of co-infection with schistosomiasis and HBV on the individual disease course

Chronic infections with hepatotropic viruses, such as HBV, can lead to liver cirrhosis, and an expected synergistic effect might exacerbate hepatic pathology during concomitant infections of HBV and schistosomiasis. Epidemiological studies were performed to investigate schistosomiasis/HBV co-infections, as no suitable animal models exist. A number of studies proposed an increased susceptibility to HBV caused by schistosomal infections (especially the severe hepatosplenic form) [46,47]. However, this could be explained by the frequent need of schistosomiasis patients for blood transfusion, especially on considering the poor infection control measures in countries where this disease is endemic. In contrast, other studies rejected the thesis stating any relationship between schistosomiasis and HBV [48,49]. Woodchucks are susceptible to infection with both schistosoma and woodchuck hepatitis virus (WHV) [50]. Because HBV and WHV descend from the same family (family *Hepadnaviridae*), a concomitant infection of schistosomes and WHV in woodchucks might be a suitable animal model for concomitant infection in humans. However, there was no impact of schistosome infection on WHV serum markers [51]. HBV replication was inhibited in transgenic mice [52] during schistosomal infection, where the antiviral effects of schistosomes related to IFN-γ and nitric oxide. Thus, most published studies have found no association between schistosomiasis and worsening of HBV infection [53]. In contrast, other studies described a poor prognosis of HBV when associated with hepatosplenic schistosomiasis [17,24,54]. Interestingly HBV vaccines (both 1st and 2nd generation) can mount an immune response in schistosomiasis patients; however, reduced responses to vaccination were seen in hepatosplenic schistosomiasis [37,55,56]. Therefore, further studies are needed to determine conclusions regarding co-infection schistosomiasis and HBV.

### Impact of co-infection with schistosomiasis and HCV on the individual disease course

*S. mansoni* infection increases HCV morbidity and chronicity, which might be referred to antecedent liver decompensation [42,57,58]. Increased HCV RNA titers, histological activity, incidence of cirrhosis/hepatocellular carcinoma, and higher mortality rates were noticed in patients with co-infection [17]. However, a sample size of 17 patients in the previous study is not big enough to draw solid conclusions. Other studies reports found no evidence that schistosomiasis affected the outcome of HCV infected patients [53,59].

### Impact of anti-schistosomal and anti-viral therapy on co-infection

Praziquantel is a first line therapy for the management and mass chemotherapy of schistosomiasis, and significantly reduced *S. mekongi* infection (from 50% to 3%) and the associated severe liver disease [60]. Because it is administered orally, praziquantel avoids the use of needles and therefore lowers the risk of HBV and HCV transmission in low-income countries. In a mouse model, praziquantel improved the immunogenicity of HBV vaccine [38]. Certain therapies used for the treatment of HBV infection have a positive effect on schistosomiasis co-infection [61]. In sixty-seven patients with advanced schistosomiasis and HBV co-infection, the use of Entecavir for 52 weeks improved liver fibrosis markers and the Ishak fibrosis score [61]. In a mouse model of infection with schistosomiasis, the use of pegylated IFN (Peg-IFN)-a drug used in treatment of HCV-significantly reduced the worm load in mice [62]. The use of Peg-IFN could be considered for use in humans to assist patients in whom the currently used oral therapy has failed.

Another important issue in this context is the effect of schistosomiasis-HCV co-infection on the response to HCV treatment where some investigators have proposed that co-infection retards response to this treatment [28]. However, Derbala et al. provided a rather more solid evidence in their clinical trial that co-infection does not affect the response to such therapy [63]. Measures to reduce the transmission of HBV and HCV by attention to hygiene and strict hygienic practices, vaccination, early detection in high-risk populations and treatment will be valuable in reducing the morbidity and mortality associated with schistosomiasis co-infection.

### Future prospects

As elimination of schistosomiasis is a global target, there is a need for supplementary tools, such as vaccination, to confer long-term prevention. Different approaches for vaccination development have been tested in animal models with variable immunogenicity including *Biomphalaria alexandrina* snail nucleoproteins, thyroid hormone receptors and more recently, the use of DNA-Sm-p80, and DNA-Sm14 [64-68]. The development of these vaccines relayed mainly on using a subunit approach where the minimal microbial constituents necessary to surmount an appropriate immune response are incorporated into the vaccine, these constituents were tegument proteins most of the times; nevertheless, despite the potential advantages of subunit vaccines, this method resulted in poorly immunogenic vaccines, a thing that entailed the co-administration with potent adjuvants, and in some cases, the addition of T helper epitopes to evoke a long lasting immune response[69].

Current approaches for HCV vaccine comprise a number of approaches of which are the adoption of recombinant E1 and E2 proteins, manufactured peptides, viral particles, viral vehicles, DNA vaccines, dendritic cells, and prime-boost strategies. Nevertheless, there are a number of problems including restricted humoral and cell mediated responses, low transmission of likely protective viral epitopes, and low efficiency of the adjuvants used in different protocols [70]. However, a greater understanding of co-infection and human trials are required to determine the mechanisms of vaccine-induced immunity, to establish a rational ground for novel vaccines and their optimal design. As a number of combined vaccines including HBV vaccine have been proven efficacious [71,72], a combination of HBV and a future schistosomiasis vaccine might also prove beneficial.

## Conclusion

There are few published data on schistosomiasis, HCV and HBV co-infection. Previous studies reported a high prevalence of HCV and HBV co-infection in countries where schistosomiasis is endemic. The effect of HBV and HCV on schistosomiasis and vice versa remains to be a matter of debate. Vaccination is the most likely method for prevention of schistosomiasis and preventing its synergistic interactions by co-infection with HBV or HCV.
